# Association Between Blood Lead Levels, Haemoglobin and Anaemia in Pregnant Women: A Register-Based Cohort Study From the Autonomous Republic of Adjara, Georgia

**DOI:** 10.1177/11786302261433111

**Published:** 2026-04-03

**Authors:** Aung Soe Htet, Tinatin Manjavidze, Nona Ephadze, Rusudan Shavishvili, Erik Eik Anda, Charlotta Rylander

**Affiliations:** 1 Department of Community Medicine, Faculty of Health Sciences, UiT, The Arctic University of Norway, Tromsø, Norway; 2 National Center for Disease Control and Public Health, Tbilisi, Georgia; 3 Adjara Public Health Center, Batumi, Georgia

**Keywords:** blood lead level, haemoglobin, anaemia, pregnant women, Adjara, Georgia

## Abstract

Many children and pregnant women in the Autonomous Republic of Adjara, Georgia experience elevated blood lead levels (BLLs) due to considerable environmental exposure from both known and unknown sources. Anaemia during pregnancy is also common in Georgia; however, the association between lead exposure and anaemia during pregnancy has not yet been assessed. This retrospective cohort study aimed to investigate the association between BLLs and haemoglobin (Hb) levels and anaemia among pregnant women. We included 3594 pregnant women who underwent BLL testing between September 2020 and July 2023. Data on Hb levels were obtained from the Georgian Birth Registry. Pregnant women were included if they had BLL testing before Hb measurements across different trimesters. The prevalence of anaemia among the participants was 23.9%, with 4.6% experiencing moderate-to-severe anaemia. Forty-two percent of pregnant women had BLLs ≥5.0 µg/dl. Each BLL increment was associated with a 0.013 g/dl (95% CI: 0.005-0.022) increase in Hb after adjusting for confounders. However, there was no statistically significant association between BLLs and the odds of moderate-to-severe anaemia (AOR for BLL ≥10 µg/dl: 0.92, 95% CI: 0.51-1.67). The weak positive association between BLL and Hb levels may therefore reflect residual confounding from unmeasured lifestyle factors such as diet and dietary supplement use.

## Background

Anaemia is characterised by reduced red blood cell or haemoglobin (Hb) levels, which decrease the blood’s oxygen-carrying capacity.^1^ Globally, anaemia affects approximately 37% (32 million) of pregnant women and 30% of all women aged 15 to 49 years.^2^ In 2021, 1.9 billion people had some form of anaemia, resulting in 52 million years lived with disability (YLDs).^3^ The most common form is iron-deficiency anaemia, which alone accounts for approximately 22% of maternal deaths globally and increases the risk of severe complications such as postpartum haemorrhage and shock, which may result in maternal death.^4^ Pregnant women with severe anaemia face a mortality risk 3.5 times greater than that for women without anaemia.^5^ Furthermore, anaemia is associated with adverse foetal outcomes, including preterm birth, small-for-gestational-age live births, and perinatal mortality.^6^

Lead is a well-documented pollutant with numerous systemic and haematological adverse health effects.^7-9^ Blood lead levels (BLLs) reflecting both present and past exposure are the most commonly used biomarker for lead exposure in humans. Lead is stored in the bones, and during pregnancy, BLLs increase as maternal bones release calcium for the foetus, concurrently releasing lead. BLLs typically decrease in the second trimester due to increased blood volume but rise again in the third trimester and may remain elevated postpartum and during breastfeeding.^10,11^

Once in the human body, lead can interfere with the absorption and utilisation of iron, essential for red blood cell production, as lead and iron compete for the same dimetal-ion transporter in the duodenum.^12^ Lead also inhibits haeme synthesis by suppressing δ-aminolaevulinic acid dehydratase (ALAD) and ferro chelatase, which results in increased urinary porphyrins, coproporphyrin, and ALAD, and increased erythrocyte protoporphyrin, ultimately reducing Hb levels.^10,13^ Lead-induced oxidative stress may further contribute to haematological damage.^10^ Additionally, as 99% of lead in the blood binds to red blood cells, this provides a pathway for toxic effects throughout the body.^10,14^ Previous studies have shown that BLLs are inversely associated with Hb levels in children^15-17^; however, the consistency and strength of this association in pregnant women remains unclear,^18-21^ likely due to varying exposure thresholds, nutritional status, and geographic contexts, underscoring the need for more population-based studies.

**Figure 1.**
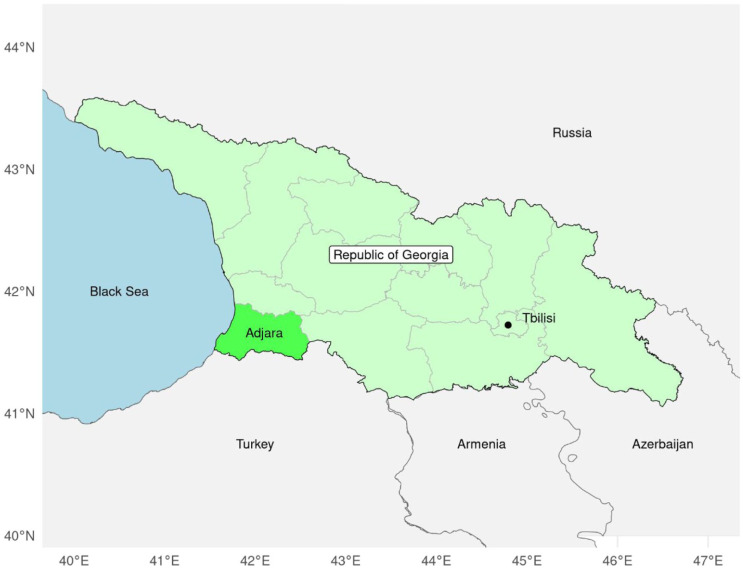
Map of the Republic of Georgia, highlighting the Autonomous Republic of Adjara.

Although most existing research on BLLs and anaemia have been conducted in high and middle-income countries, little data exist for Eastern Europe and the Caucasus region. In Georgia, the 2018 Multiple Indicators Cluster Survey (MICS) reported that 41% of children aged 2 to 7 years had BLLs ≥5 µg/dl, with this figure increasing to 85% of the children in West Georgia’s Autonomous Republic of Adjara.^22^ In 2020, the government of the Autonomous Republic of Adjara (Figure 1) implemented a lead biomonitoring programme for pregnant women as an integrated part of the antenatal care (ANC) programme. A recent Adjara Region study found that 40% of women in their first trimester of pregnancy had BLLs ≥3.5 µg/dl in 2023, with a 59% drop in mean BLLs among all pregnant women from 2020 to 2023.^23^ However, despite the recent decline in BLLs among pregnant women in Adjara, elevated BLLs continue to pose a significant public health challenge in the region. Over the years, some sources of lead exposure have been identified in Georgia, though many remain unknown. High concentrations of lead have been detected in spices^24^; however, stricter regulations, awareness campaigns, and improved product controls appear to have mitigated this issue.^23,25^ Other likely sources of lead exposure include old paint, which is prevalent in Georgia, as well as painted ceramics.^26,27^ Additionally, mining—a key sector of Georgia’s economy—may contribute to environmental challenges due to the extraction of minerals such as manganese, copper, gold, and coal.^28^ Scrap metals containing lead are also widespread in the Georgian environment and may further contribute to population lead exposure.

Despite these concerns, little is known about the association between anaemia and BLLs in Georgia, particularly among pregnant women. BLLs can vary based on socioeconomic status, nutrition, and overall health—factors that may also influence Hb levels. No BLL is considered safe for pregnant women,^10,29^ and even low BLLs remain a potential public health problem.

To address these knowledge gaps, this study aimed to assess anaemia prevalence and investigate the association between BLLs and Hb/moderate-to-severe anaemia among pregnant women in the Autonomous Republic of Adjara, Georgia. This is the first study from Adjara to examine the association between lead exposure during pregnancy and Hb on population level.

## Methods

The Georgian Birth Registry (GBR) is a statutory health registry recording all childbirths and ANC visits at medical facilities across Georgia. The GBR collects detailed demographic, medical history, and pregnancy-related data, including Hb levels. The National Centre for Disease Control and Public Health (NCDC) of Georgia merged lead screening results from Adjara with GBR data using unique citizen identification numbers.

### Study Design and Population

This retrospective cohort study analysed BLL data collected from September 2020 to July 2023 as part of the Adjara biomonitoring programme for pregnant and breastfeeding women. During this period, 10 116 pregnant women underwent at least 1 BLL test during pregnancy or postpartum.

For this study, we applied the following inclusion criteria:

- Pregnant women who had BLL testing during pregnancy
- Availability of Hb measurements in subsequent trimesters
- Temporal precedence: BLL testing conducted before Hb measurements.

Exclusion Criteria:

- BLL tests performed outside pregnancy or postpartum
- Non-pregnant women or those residing outside Adjara
- Concurrent BLL and Hb measurements within the same trimester
- Incomplete Hb data, including;
  ○ Second-trimester BLL measurements with only first-trimester Hb measurements
  ○ Third-trimester BLL measurements without complete Hb data
  ○ No Hb measurements during ANC

**Figure 2.**
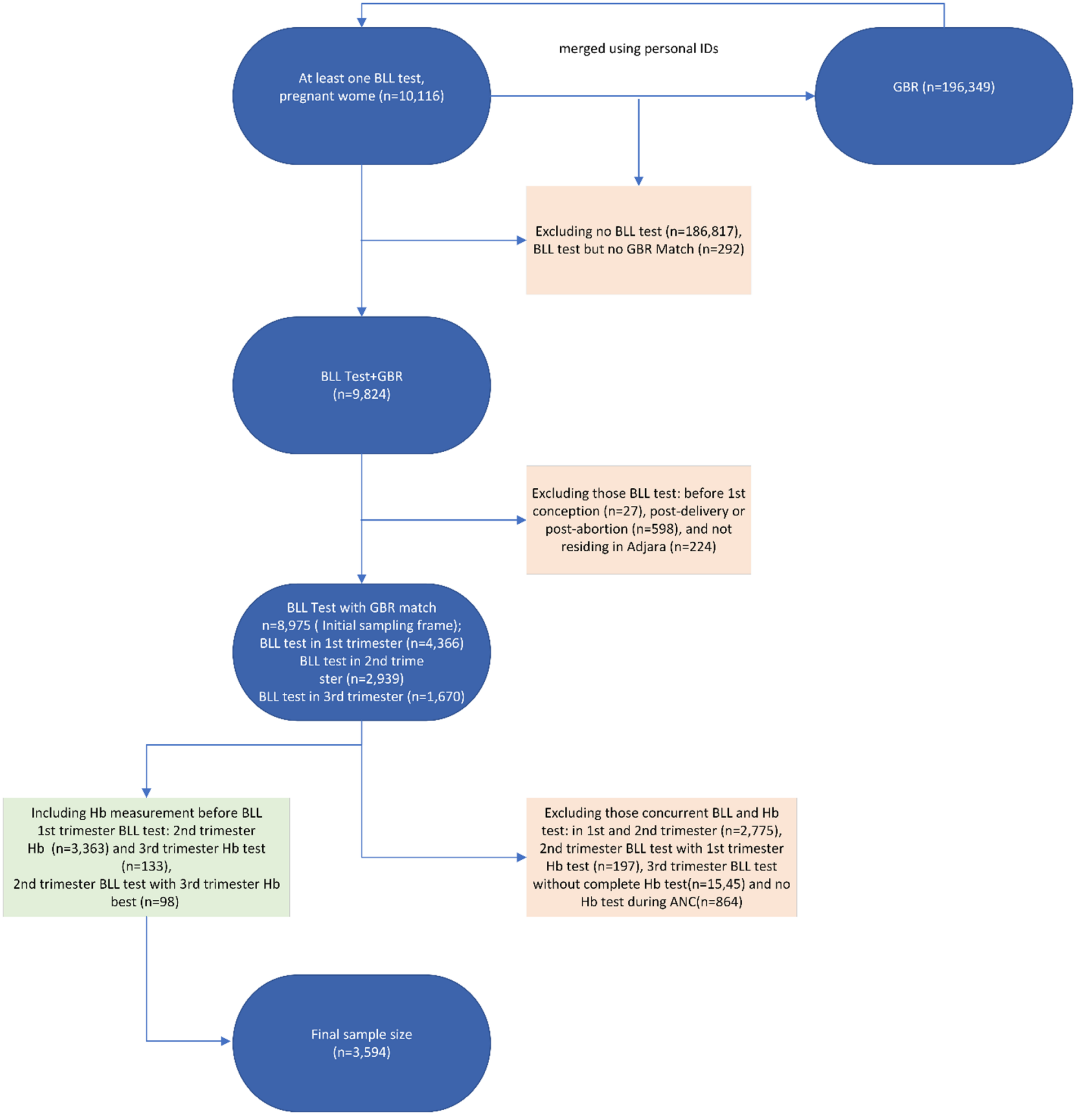
Flow chart of the sampling procedure: data from the lead biomonitoring programme of pregnant women in Adjara and the Georgian birth registry, 2020 to 2023.

After applying these criteria, the initial sample of 10 116 women was reduced to 8975 (Figure 2). From this cohort, 3594 women met all study design requirements and had BLL and Hb data:

- 3363 women with first-trimester BLL and second-trimester Hb measurements.
- 133 women with first-trimester BLL and third-trimester Hb measurements.
- 98 women with second-trimester BLL and third-trimester Hb measurements.

### BLL Testing

During ANC visits, 6 ml of venous blood was collected in EDTA tubes for trace element analysis and transported to Mediprime LLC Medical Laboratory (ISO15189), Tbilisi, Georgia. Initially, 100 µl of whole blood was placed into an Eppendorf tube. To this mixture, 100 µl of 2% ultrapure nitric acid (HNO3, 70%, redistilled, ≥99.999% trace metals basis, MERCK) and 900 µl of a modifier solution were added. The mixture was then vortexed at 5000 rpm for 1 minute. Then, 300 to 500 µl of the supernatant was extracted and analysed using a graphite furnace Agilent 280Z Atomic Absorption Spectrometer equipped with Zeeman background correction (Agilent Technologies, California, USA).

The modifier solution consisted of 400 ml of deionised water, 25 ml of TRITON™ X-100 (v/v) 10% solution (J. T. Baker), 5 ml of a 20% ultrapure ammonium dihydrogen phosphate (NH4H2PO4) solution (99.9%, trace metals basis, Thermo Scientific), and 1 ml of concentrated HNO3, which was added to 500 ml of deionised water. Blank samples of whole blood (blank control, ACQ Science) and reference material samples (Seronorm™Trace Element serum, Sero) were processed using the same method parallel with the blood samples. The absolute concentration of the certified reference material did not deviate more than +/− 10% from the certified values. The method detection limit (MDL) was 0.9 µg/dl.

Hb measurements were performed during ANC visits to designated ANC clinics with data registered in GBR.

### Variables

Pregnant women were categorised into anaemia groups based on the first Hb measurements recorded during pregnancy. Anaemia was categorised by trimester-specific Hb levels: normal (Hb ≥11.0 g/dl in the first and third trimesters, and ≥10.5 g/dl in the second trimester), mild anaemia (Hb ranges from 10.0 to 10.9 g/dl in the first and third trimesters, and 9.5 to 10.4 g/dl in the second trimester), and moderate-to-severe anaemia ( Hb <10.0 g/dl in the first and third trimesters, and <9.5 g/dl in the second trimester).^1^

We categorised pregnant women into 4 lead exposure groups based on their first BLL measurement during pregnancy; BLL <3.5 µg/dl, 3.5 g/dl ≤ BLL < 5.0, and 5.0 ≤ BLL <10.0 µg/dl, and BLL ≥10.0 µg/dl, following United State CDC and the World Health Organisation (WHO)—aligned public health thresholds,^30,31^ where <3.5 µg/dl reflects the CDC reference value and ≥5 µg/dl indicate levels requiring follow-up or clinical intervention.

Educational level was classified into 4 categories: higher, secondary, primary, or unknown which corresponds to the highest education completed at the time of pregnancy. Residency was classified as either rural or urban. Parity was divided into nulliparous (no children) and multiparous (≥1 child). ANC visits were grouped into <4, 4-8, and >8 visits. Body mass index (BMI) at the first ANC visit was categorised into underweight (BMI <18.5 kg/m^2^), normal weight (BMI 18.5-24.9 kg/m^2^), overweight (BMI 25.0-29.9 kg/m^2^), and obesity (BMI ≥30.0 kg/m^2^).

### Statistical Analysis

**Figure 3.**
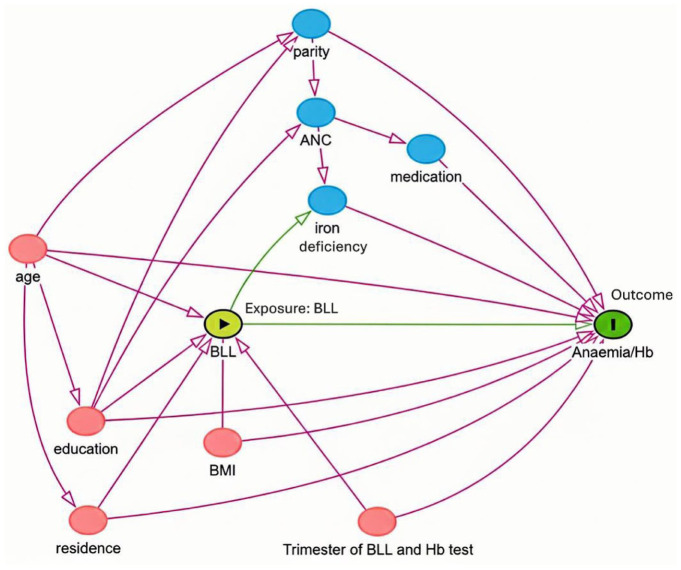
DAG illustrates potential confounders and mediators in the association between BLLs and Hb/moderate-to-severe anaemia. Pink circles represent confounding factors, whereas blue circles represent mediating factors. Green lines denote causal pathways, and red lines indicate biasing (non-causal) pathways.

Descriptive analyses estimated anaemia prevalence according to maternal characteristics and BLL groups, with results presented as frequencies, percentages, means, geometric means and standard deviations (SDs). For Hb and BLL, we used 1-way analysis of variance (ANOVA) to assess differences between anaemia groups while chi-squared tests were performed to test the differences between the categorical groups. A directed acyclic graph (DAG) was used to identify confounders, mediators, and colliders in order to investigate the presumed causal association between BLLs and anaemia/Hb (Figure 3). Based on the DAG, we adjusted for age, education, residency, trimester of blood tests (BLL and Hb) and BMI at the first ANC visit in order to estimate the total effect of BLLs on Hb /anaemia status. Hence, as our aim was to estimate the total effect, mediators were not included in the regression models. Linear regression was used to assess the association between BLLs and Hb, while logistic regression models were employed to calculate odds ratios (ORs) with 95% confidence intervals (CIs) for the association between BLLs and moderate-to-severe anaemia. The final analysis included 2 models: Model 1 (crude) and Model 2 (adjusted for the confounders listed above). Logistic regression analysis modelling BLL as a continuous measure was used to estimate the linear trend (*P* trend). To assess non-linearity, we included a natural cubic spline with 4 knot in the regression models. There was no evidence of non-linearity; therefore, the results presented are from linear models. Missing data for covariates included in the analysis (eg, education, BMI) were handled using listwise deletion, as the proportion of missing values was very low (less than 1%). Robustness checks were performed to ensure the reliability of the results. These included sensitivity analyses excluding participants with high BMI or third-trimester Hb values, which could act as potential outliers or confounders, as well as multicollinearity diagnostics. No significant interactions were found between BLL and covariates, including age, education, residency, trimester of blood tests (BLL and Hb) and BMI at the first ANC visit. Statistical analyses were conducted using Stata/MP version 18.0 (StataCorp, TX, USA). Statistical significance was set at *P* < .05.

## Results

Among the 3594 pregnant women, 23.9% had anaemia, including 19.3% with mild anaemia, and 4.6% with moderate-to-severe anaemia (Table 1). The mean Hb level among the study participants was 11.1 g/dl (SD = 0.95), the mean BLL was 5.31 µg/dl (SD = 3.67), and the geometric mean BLL was 4.28 µg/dl (geometric SD = 1.97). Additionally, 32.7% had a BLL between 5.0 and 10 µg/dl, and 9.5% had a BLL ≥10 µg/dl (not shown in the tables). Most participants were aged 25 to 34 years (58.2%), resided in urban areas (65.6%), had normal weight (52.8%), had 4 to 8 ANC visits (52.5%), and were multiparous (60.5%; Table 1). Approximately 47% of the participants reported secondary school as their highest completed educational level.

**Table 1.** Characteristics of Study Subjects by Anaemia Prevalence in Adjara (2020-2023) n = 3594.

Characteristics	Total	No anaemia	Mild anaemia	Moderate-to-severe anaemia	*P*-value^c^
n (row%)	3594 (100)	2736 (76.1)	693 (19.3)	165 (4.6)	
Hb, mean g/dL (SD)	11.1 (0.95)	11.47 (0.69)	10.07 (0.28)	8.96 (0.52)	<.001
BLL, mean µg/dl (SD)	5.31 (3.67)	5.42 (3.74)	4.91 (3.44)	5.32 (3.57)	.045
BLL, GM µg/dl (SD)	4.28 (1.97)	4.37 (1.97)	3.95 (1.98)	4.34 (1.93)	.038
Age group, n (%)					
15-24	972 (27.1)	713 (26.1)	206 (29.7)	53 (32.1)	.057
25-34	2092 (58.2)	1609 (58.8)	397 (57.3)	86 (52.1)	
>35	530 (14.7)	414 (15.1)	90 (13.0)	26 (15.8)	
Education level, n (%)					
Higher	1183 (32.9)	920 (33.6)	219 (31.6)	44 (26.7)	.203
Secondary	1679 (46.7)	1270 (46.4)	327 (47.2)	82 (49.7)	
Primary	61 (1.7)	49 (1.8)	7 (1.0)	5 (3.0)	
Unknown	671 (18.7)	497 (18.2)	140 (20.2)	34 (20.6)	
Residence, n (%)					
Urban	2359 (65.6)	1750 (64.0)	486 (67.9)	123 (74.5)	<.001
Rural	1235 (34.4)	986 (36.0)	207 (29.9)	42 (25.5)	
BMI at first ANC visit, n (%) ^a^					
Underweight	211 (5.9)	142 (5.2)	59 (8.5)	10 (6.1)	<.001
Normal	1894 (53.0)	1398 (51.2)	387 (55.8)	109 (66.5)	
Overweight	897 (25.1)	719 (26.4)	147 (21.2)	31 (18.9)	
Obese	584 (16.4)	470 (17.2)	100 (14.4)	14 (8.5)	
Parity, n (%)^b^					
Nulliparous	1261 (40.8)	970 (40.0)	231 (37.5)	60 (39.5)	.530
Multiparous	1933 (59.2)	1456 (60.0)	385 (62.5)	92 (60.5)	
Number of ANC visit, n (%)					
1-3	2 (0.06)	2 (0.1)	0 (0.0)	0 (0.0)	.236
4-8	1886 (52.5)	1459 (53.3)	352 (50.8)	75 (45.5)	
8+	1706 (47.5)	1275 (46.6)	341 (49.2)	90 (54.5)	
Year of study, n (row%)					
2020	178 (4.9)	142 (79.8)	30 (16.9)	6 (3.4)	.002
2021	1275 (35.5)	922 (72.3)	274 (21.5)	79 (6.2)	
2022	1604 (44.6)	1260 (78.6)	283 (17.6)	61 (3.8)	
2023	537 (15.0)	412 (76.7)	106 (19.7)	19 (3.5)	

Abbreviations: ANC, antenatal care; BLL, blood lead levels; BMI, body mass index; GM, geometric mean; Hb, haemoglobin; SD, standard deviations.

a Missing respondents - 8.

b Missing respondents – 400.

c Chi-squared tests were performed for each characteristic. ANOVA was used for mean Hb and BLL comparison.

There were statistically significant differences in BLLs across anaemia groups (*P* = .045). Women with moderate-to-severe anaemia were more likely to be 15 to 24 years (32.1%) compared to those without anaemia (26.1%) or with mild anaemia (29.7%), however the difference was not statistically significant. No difference was observed in the education level. Women with moderate-to-severe anaemia were significantly more likely to reside in urban areas (74.5%) than without anaemia (64.0%). Women with moderate-to-severe anaemia were significantly more likely to be normal body weight (66.5%) compared to those without anaemia (51.2%) or mild anaemia (55.8%). No difference was observed in the number of ANC visits across the anaemia groups. The prevalence of moderate-to-severe anaemia increased from 3.4% in 2020 to a peak of 6.2% in 2021, followed by a steady decline to 3.8% in 2022 and 3.5% in 2023.

The prevalence of anaemia across BLL categories showed no statistically significant differences (*P* = .101; Table 2). Women with BLL 3.5 - <5.0 µg/dl had the highest prevalence of moderate-to-severe anaemia (5.1%), which was comparable to the prevalence among women with BLL ≥ 10 µg/dl (4.7%).

**Table 2.** Prevalence of Anaemia Groups Across BLL Categories in Pregnant Women in Adjara (2020-2023), n = 3594.

	No anaemia	Mild anaemia	Moderate-to-severe anaemia	*P*-value^a^
BLL category	n (row %)	n (row %)	n (row%)	
<3.5 µg/dl	986 (74.4)	279 (21.0)	61 (4.6)	
3.5-<5.0 µg/dl	556 (74.0)	157 (20.9)	38 (5.1)	
5.0-<10.0 µg/dl	922 (78.5)	202 (17.2)	50 (4.3)	
≥10 µg/dl	272 (79.3)	55 (16.0)	16 (4.7)	.101

Abbreviation: BLL, blood lead levels.

a Chi-squared test was performed to assess the association between anaemia groups and BLL categories.

For each 1 µg/dl increase in BLL, the crude linear regression model showed a weak but statistically significant increase in Hb levels of 0.012 g/dl (95% CI: 0.003-0.020; Table 3). After adjustment for age, education, residency, trimester of blood test and BMI at the ANC visit, the coefficient slightly increased to 0.013 g/dl (95% CI: 0.005-0.022).

**Table 3.** Association between BLLs and Hb Levels (Linear Regression).

Description	n	Crude coefficient (95% CI)	n	Adjusted coefficient (95% CI)^a^
BLL (continuous)	3594	0.012 (0.003-0.020)	3586	0.013 (0.005-0.022)

Abbreviations: ANC, antenatal care; BLL, blood lead levels; BMI, body mass index: Hb, haemoglobin.

a Adjusted for age, education, residency, trimester of blood test, and BMI at ANC visit.

There was no significant association between BLL categories and the odds of moderate-to-severe anaemia (Table 4). Compared to the reference group (BLL < 3.5 µg/dl), the 3.5 - <5.0 µg/dl group showed non-significant increase in odds of moderate-to-severe anaemia (crude OR = 1.10, 95% CI: 0.73-1.67; adjusted OR = 1.10, 95% CI: 0.71-1.71). In contrast, the 5.0 - <10.0 µg/dl group showed non-significant decreased odds (crude OR = 0.92,95% CI: 0.63-1.35; adjusted OR = 0.78, 95% CI: 0.52-1.19), while the highest BLL category (≥10 µg/dl) had near-null associations (crude = 1.01, 95% CI: 0.58-1.78; adjusted OR = 0.92, 95% CI: 0.51-1.67). There was no linear trend between BLL and the odds of moderate-to-severe anaemia (*P* = .779.)

**Table 4.** Association Between BLL Categories and Moderate-to-Severe Anaemia (Logistic Regression).

BLL category	n	Crude OR (95% CI)	Adjusted OR (95% CI)^a^
<3.5 µg/dl (ref.)	1326	1.00	1.00
3.5 - <5.0 µg/dl	751	1.10 (0.73-1.67)	1.10 (0.71-1.71)
5.0 - <10.0 µg/dl	1174	0.92 (0.63-1.35)	0.78 (0.52-1.19)
≥10 µg/dl	343	1.01 (0.58-1.78)	0.92 (0.51-1.67)

**Table:** Trend analysis (BLL continuous).

Variable	n	Crude OR (95% CI)	n	Adjusted OR (95% CI)^a^	*P*-value
BLL (continuous)	3594	1.00 (0.96-1.04)	3586	0.99 (0.95-1.04;0.779)	.779

Abbreviations: ANC, antenatal care; BLL, blood lead levels; BMI, body mass index.

a Adjusted for age, education, residency, trimester of blood test, and BMI at ANC visit.

## Discussion

### Principal Findings

In our population-based cohort from Adjara, Georgia, 24% of pregnant women had anaemia at their first Hb measurement during ANC, with 4.6% suffering from moderate-to-severe anaemia. Mean BLL was 5.32 µg/dl clearly indicating that elevated BLL remains a public health concern in Adjara. We observed a weak positive association between BLL and Hb, but BLL categories were not associated with the odds of moderate-to-severe anaemia. Furthermore, there was no significant linear trend between increasing BLL and moderate-to-severe anaemia. Hence, from our findings, it seems unlikely that the current lead exposure situation increases the risk of clinically relevant anaemia among pregnant women attending ANC in Adjara.

### Comparisons With Previous Studies

The overall anaemia prevalence was lower than the global estimate of 36%,^2^ and the prevalence of moderate-to-severe anaemia was also lower than the estimates for Central and Eastern Europe (8%) and the global estimate (17%).^2^ These differences may partly be explained by the updated WHO trimester-specific Hb thresholds which was applied in the current study, which set lower thresholds in the second trimester than previously.^1^ It may also be explained by the high ANC attendance (≥4 visits by 99% of participants) in Adjara and free access to iron supplements in Georgia during ANC, if needed. Mean BLL (5.31 µg/dl) was greater than those reported in pregnant women in Turkey (2.8 µg/dl)^32^ and Mexico (2.79 µg/dl) in 2008,^18^ but remained below the global pooled mean value (6.85 µg/dl).^33^ Notably, 42% had BLLs ≥ 5 µg/dl, levels at which the WHO recommends clinical intervention for pregnant women.^30^ Elevated BLLs among participants may result from short- and long-term environmental lead exposure in Adjara, from for instance, illegally adulterated spices, previous use of leaded gasoline, leaded paint, painted ceramics and a large number of other possible sources.^24,34,35^ Encouragingly, there was a significant reduction in average BLLs among pregnant women in Adjara from 2021 to 2023.^23^

The weak but positive association between BLL and Hb observed in this study aligns with finding from a cohort study in China (n = 1151).^20^ Other studies on pregnant women have shown mixed results: a cross-sectional study in Mexico (n = 292) reported a non-significant positive association between BLL and Hb levels,^18^ while 1 in Jordan (n = 167) found no association.^36^ In contrast, studies on pregnant women in Brazil (n = 55) and India (n = 140),^19,37^ and children in China (n = 743), Philippines (n = 2861), and Pakistan (n = 340) reported inverse associations between BLLs and Hb levels.^15,16,38^ The studies differ in target population, design, sample size, measurements and confounding control which may impact their results.

The positive association between BLLs and Hb in this study should be interpreted the presumed competition between iron and lead in the intestines in mind.^12^ In fact, it seems unlikely that our findings reflect a causal relationship between BLLs and Hb. It is more likely that the positive association can be explained by residual confounding, for instance, diet and dietary supplement, which we were unable to control for. A systematic review and meta-analysis demonstrated that increased calcium and iron intake was significantly associated with reduced BLLs in pregnant women^39^ and dietary intake can also impact anaemia development during pregnancy.^40^ Moreover, calcium supplementation has been shown to reduce BLLs among pregnant women^41,42^ and the WHO recommends calcium supplements for pregnant women with BLLs ≥5 µg/dl.^30^ If our participants had received calcium supplements during ANC visits before Hb or BLL testing due to suspected or previously measured elevated BLLs, this could have altered the strength and direction of the association, as calcium can impact both iron absorption and BLLs. Unfortunately, there was no data on the dietary intake or supplement use in this study.

Moreover, studies linking very high (BLLs >40 µg/dl) with increased anaemia risk in adults,^43^ and BLLs ≥10 µg/dl with mild to severe anaemia in children^44,45^ further suggest that associations may vary by exposure range and target population. In our study, we observed no association between various BLL categories and the odds of moderate-to-severe anaemia. However, few women had strongly elevated BLLs (>20 µg/dl), which could partly explain the discrepancies with other studies. Differences in results could also be explained by the access to free-of-charge ANC which might manage anaemia well. Unfortunately, we had no data of women not attending ANC, neither on their Hb levels or their BLLs. Hence, the associations between BLLs and Hb/anaemia in this group, remains unknown and it might also explain the results, this group of women usually has higher risks.

### Strengths and Limitations

One strength of our study is the use of the GBR, which covers nearly 100% of deliveries in Georgia and provides comprehensive demographic and medical data. The lead biomonitoring programme in Adjara offers a unique opportunity to study high-quality BLL measurements in pregnant women. To our knowledge, this is the first population-based study in Georgia to explore the association between BLLs and Hb levels and moderate-to-severe anaemia, contributing valuable data to inform public health strategies. However, our study had several limitations. One of the limitations was the variability in Hb measurement methods and instruments across ANC clinics, with no verification of exact techniques, which may have introduced Hb measurement inconsistencies. Moreover, 5381 participants who did not meet the cohort design criteria were excluded, and we had no information about women not attending ANC. This may have introduced selection bias. Even with DAG and control for confounders, residual confounding remains possible, as factors such as dietary intake, supplements and lifestyle exposures affecting BLL and anaemia were not included. Additionally, we lacked exact information on the exact timing between BLL testing and Hb measurements, which may have introduced variability in the exposure-outcome interval and limited our ability to adjust for it.

## Conclusions

No association was observed between BLLs and increased odds of moderate-to-severe anaemia, however, we observed a weak but significant positive association between BLL and Hb levels. This association is likely explained by residual confounding by unmeasured nutritional or lifestyle factors. With 24% of the pregnant women in Adjara had anaemia, and 42% exceeded the WHO’s intervention thresholds for BLLs (≥5.0 µg/dl), lead exposure remains a significant public health concern. Continued lead screening for pregnant women should be encouraged for 2 key reasons: (a) exposure remains relatively high, increasing the likelihood of identifying pregnant women with elevated BLLs who can be informed about reducing lead exposure, and (b) the existing screening system provides a valuable opportunity to conduct further research on lead exposure in pregnant women, benefitting the population as a whole. Additionally, public health campaigns to raise awareness about potential lead exposure sources and the importance of a healthy diet could further contribute to improved maternal health.
